# Effect of Nutritional Factors on Mental Health Problems in Autism Spectrum Disorder: A Narrative Synthesis of Current Evidence

**DOI:** 10.1002/brb3.71568

**Published:** 2026-07-08

**Authors:** Nalan Hakime Nogay, Gulin Ozturk Ozkan

**Affiliations:** ^1^ Department of Nutrition and Dietetics Faculty of Health Sciences Bursa Uludag University Bursa Turkey; ^2^ Department of Nutrition and Dietetics Faculty of Health Sciences Istanbul Medeniyet University Istanbul Turkey

**Keywords:** autism spectrum disorder, dietary intervention, gut–brain axis, mental health, nutrition, omega‐3 fatty acids, probiotics, vitamin D

## Abstract

**Background:**

Autism spectrum disorder (ASD) is frequently associated with co‐occurring mental health challenges, such as anxiety and irritability, which severely impair quality of life. While nutritional research typically targets core ASD symptoms, this narrative synthesis specifically investigates the impact of nutritional interventions on these psychiatric comorbidities.

**Methods:**

A structured literature search was performed across PubMed, Web of Science, and Scopus databases. Twenty‐one peer‐reviewed studies met the inclusion criteria, focusing on the relationship between dietary interventions/supplements and mental health outcomes in ASD populations.

**Results:**

Findings regarding gluten‐free and casein‐free (GFCF) diets remain inconclusive due to highly mixed results. While some trials involving vitamin D3, omega‐3 fatty acids, and probiotics have reported improvements in anxiety and irritability, these findings are characterized by significant inconsistency. The overall strength of the evidence is limited by small sample sizes, short intervention durations, heterogeneous dosage protocols, and the use of diverse behavioral assessment scales.

**Conclusions:**

Vitamin D3, omega‐3 fatty acids, and probiotics show preliminary signals of benefit for managing certain mental health symptoms in ASD; however, current data are insufficient to confirm clinical effectiveness or to support the development of formal clinical guidelines. Future research requires large‐scale, randomized, double‐blind, placebo‐controlled trials with standardized outcome measures and longer follow‐up periods to clarify these inconsistent findings.

## Graphical abstract

This narrative synthesis of 21 studies evaluates nutritional interventions for psychiatric comorbidities in ASD. Vitamin D3, omega‐3 fatty acids, and probiotics show preliminary benefits for anxiety and irritability, whereas GFCF diet results remain inconclusive. Methodological limitations preclude formal clinical guidelines, emphasizing that individualized balanced nutrition remains the clinical priority.

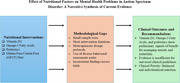

## 1 Introduction

It is well‐established that individuals with autism spectrum disorder (ASD) have an increased risk of experiencing co‐occurring mental health problems, a situation that negatively impacts the quality of life for both the individuals with ASD and their caregivers (Lai et al. 2019; Curnow et al. 2023). The prevalence of accompanying mental health problems is approximately 70% in children with ASD, while this rate ranges between 91%–95% in adolescents and adults with ASD (Backman et al. 2023; Hossain et al. 2020). A meta‐analysis conducted by Lai et al. (2019) determined that the prevalence of anxiety disorders is 20% and depression is 11% among individuals with ASD. Another study on this topic found that anxiety disorders occurred in 42.0%, oppositional defiant disorder (ODD) in 29.0%, and attention deficit hyperactivity disorder (ADHD) in 22.6% of children with ASD aged 2–17 years (Halvorsen et al. 2025).

Diagnosing mental health problems in ASD is often challenging because these issues frequently share similarities with core ASD symptoms. As a result, it is not easy to determine whether these manifestations are indicative of a true mental illness or merely a reflection of repetitive and restricted behaviors and interests (Jasim and Perry 2023). Furthermore, difficulties in expressing thoughts and emotions, and/or the presence of co‐occurring intellectual disability, can complicate self‐reporting (Halvorsen et al. 2025). Symptoms such as repetitive and ritualistic behaviors are observed to increase in autistic individuals experiencing anxiety. All these factors collectively make the diagnosis of mental illness difficult in this population (Helverschou and Martinsen 2011).

Internalizing problems such as anxiety, depression, and excessive fear are among the most frequently observed mental health issues in individuals with ASD. It is hypothesized that internalizing problems are either a cause of ASD symptoms (e.g., rigid preferences for sameness resulting from excessive anxiety) or an effect (e.g., communication difficulties leading to isolation and anxiety) (Tick et al. 2016). One study reported a strong correlation between communication difficulties and repetitive/restrictive behaviors with generalized anxiety and negative affect (Hallett et al. 2012). Behaviors commonly seen in ASD, such as verbal rituals and repetitive questioning, can also be interpreted as symptoms of anxiety. All these factors suggest that the core symptoms of ASD can alter the clinical presentation of mental health problems (Lecavalier et al. 2014). It has been reported that behavioral problems and ADHD, which are common in childhood among individuals with ASD, tend to decrease during adolescence, but anxiety and depression show an increase (Mukherjee and Beresford 2023).

Factors influencing mental health problems in children and adolescents with ASD include multicentric factors such as cognitive factors, socio‐environmental factors, and adaptive functioning skills (Mukherjee and Beresford 2023). Nutrition is one of these environmental factors, and most studies on its impact on ASD have focused primarily on behavioral problems. However, because the core symptoms of ASD and mental health problems are similar, the effect of nutrition on either behavioral problems or mental health issues has not been clearly delineated. This study aims to clarify the effect of nutritional factors on mental health problems observed in ASD within the framework of the current literature.

## 2 Mental Health Problems Observed in ASD and Assessment Tools

Mental health issues commonly observed in individuals with ASD include ADHD and hyperactive type behaviors, along with Tourette syndrome, depression, anxiety disorder, ODD, and obsessive compulsive disorder (OCD) (Colvert et al. 2022). Furthermore, phobias, tics, eating disorders, and schizophrenia can also occur. Several studies have reported that many individuals with ASD have more than one additional psychiatric comorbidity (Leyfer et al. 2006). It is recommended that several principles be followed when assessing mental health issues in children and adults with ASD: allocating sufficient time for the evaluation, conducting the assessment in a familiar environment, gathering information from multiple sources such as parents, teachers, and siblings, observing behavior and interaction in various settings, utilizing standardized assessment instruments, and directly learning the psychiatric/social/developmental history (Xenitidis et al. 2007).

For the initial assessment of mental health problems in individuals with ASD, the use of relatively broad‐scope standardized instruments is suggested (Halvorsen et al. 2023). It is considered more appropriate to use a scale specifically designed for behaviors unique to individuals with ASD or developmental disabilities. If ASD alters the typical clinical presentation of behavioral syndromes, using a population‐specific assessment tool will yield much more accurate information (Lecavalier et al. 2014). However, very few tools have been developed for children and adolescents with intellectual disabilities, and more information is needed regarding valid and standardized instruments to measure mental health problems (Halvorsen et al. 2023).

Among the global rating scales are the Clinical Global Impression Scale (CGI) and the Developmental Disabilities Modification of the Children's Global Assessment Scale (DD‐CGAS) (National Institute of Mental Health 1976; Wagner et al. 2007). Broad‐scope scales used in young children with ASD include the Aberrant Behavior Checklist (ABC), the Nisonger Child Behavior Rating Form (NCBRF), the Child Behavior Checklist (CBCL), the Developmental Behavior Checklist (DBC), and the ASD‐Co‐occurring Conditions (ASD‐CC) scale (Aman et al. 1996; Matson et al. 2009). The Child Symptom Inventory–4 (CSI‐4) is a broad‐scope measure based on the DSM‐IV that contains subscales representing the most frequently encountered childhood disorders (Lecavalier et al. 2014).

For identifying mental health problems in ASD, there are also narrow‐scope measurement tools that focus on a specific condition, such as anxiety or ADHD. The Yale‐Brown Obsessive Compulsive Scale Modified for Pervasive Developmental Disorders (CYBOCS‐PDD), Pediatric Anxiety Rating Scale (PARS), Research Units on Pediatric Psychopharmacology, Multidimensional Anxiety Scale for Children (MASC), and the Swanson Nolan and Pelham (SNAP) for ADHD symptoms are among the narrow‐scope rating scales (Lecavalier et al. 2014; Scahill et al. 2006; Research Units on Pediatric Psychopharmacology Anxiety Study Group 2002; March et al. 1997; Swanson 2012). Another scale, the Reiss Scales for Children's Dual Diagnosis (RSCDD), is used to screen for psychopathology in children and adolescents with dual diagnoses, such as intellectual disability and mental health disorder (Reiss and Valenti‐Hein 1994).

## 3 Impact of Nutritional Factors on Mental Health Issues

The structure, function, and composition of the brain depend on the presence of essential nutrients, including lipids, amino acids, vitamins, and minerals. Therefore, nutrient intake can affect brain functions (Adan et al. 2019). Nutrition also plays a crucial role in modulating stress and inflammation and preserving cognitive function by influencing endogenous hormones, neuropeptides, neurotransmitters, and the microbiota–gut–brain axis (Muscaritoli 2021). The interaction between social and environmental factors, nutrition, and genetic profile is thought to affect cellular and chemical mechanisms, thereby influencing the regulation of brain functions and contributing to the etiopathogenesis of mental health problems (Borges‐Vieira and Cardoso 2023). While some studies have shown that vitamin D, vitamin K, vitamin E, vitamin C, folic acid, thiamine, pyridoxine, vitamin B12, iron, magnesium, zinc, omega‐3 fatty acids, probiotics, and prebiotics have a positive effect on mental disorders such as depression, anxiety, and ADHD, differences in intervention duration, dosage used, and sample sizes across studies necessitate further research (Dionisie et al. 2025; Renteria et al. 2024; Hashim et al. 2025; Ding and Zhang 2022; Borges‐Vieira and Cardoso 2023; Mikkelsen et al. 2016). The potential mechanisms of action of these nutrients on mental health are provided in Table 1.

**TABLE 1:** Possible mechanisms of the effects of certain nutrients on mental health.

Nutrient	Mental health problem	Potential mechanism	Authors/year
Vitamin D	Depression, anxiety disorders, ADHD	These potential mechanisms include the prevention of neuroinflammation, reduction of oxidative stress, regulation of neuronal calcium (Ca_2_+) levels (which become neurotoxic at high concentrations), regulation of serotonin production, the ability to influence gut serotonin synthesis either directly by altering the gut microbiota or indirectly by enhancing gut barrier integrity or immune response, and the possession of pro‐neurogenic and neuromodulatory properties.	Dionisie et al. 2025 Renteria et al. 2024 Kouba et al. 2022 Kotsi et al. 2019
Vitamin K	Depression	Antioxidant and anti‐inflammatory effects, improvement of neuroplasticity. Furthermore, vitamin K is essential for the γ–carboxylation of Gas6. Gas6 is a protein that interacts with the TAM receptor tyrosine kinase family (Tiro3, Axl, and MerTK), which is effective in regulating various physiological processes such as neuroinflammation, myelin homeostasis, microglial regulation, and myelination.	Hashim et al. 2025
Vitamin E	Anxiety, depression	It reduces inflammation and oxidative stress.	Muscaritoli 2021 Ding and Zhang 2022
Vitamin C	Depression	It reduces oxidative stress.	Ding and Zhang 2022
Folic acid	Depression	It regulates neurotransmitter production and reduces inflammation. It acts as a cofactor in methionine–homocysteine metabolism.	Muscaritoli 2021 Borges‐Vieira and Cardoso 2023
Thiamin	Depression	It is involved in the formation of nerve impulses and the synthesis of neurotransmitters.	Mikkelsen et al. 2016
Pyridoxine	Depression	It aids in the conversion of tryptophan to niacin or serotonin. It is involved in the synthesis of GABA, dopamine, norepinephrine, and serotonin. It acts as a cofactor in methionine–homocysteine metabolism.	Mikkelsen et al. 2016 Borges‐Vieira and Cardoso 2023
Vitamin B12	Depression	It ensures the synthesis of new cells and the preservation of nerve cell function. It acts as a cofactor in methionine–homocysteine metabolism.	Mikkelsen et al. 2016 Borges‐Vieira and Cardoso 2023
Iron	ADHD	It is an essential cofactor for tyrosine hydroxylase, which is required for dopamine synthesis.	Robberecht et al. 2020
Magnesium	Anxiety, depression, ADHD	It reduces inflammation and oxidative stress and increases cell membrane stability. It is involved in the apoptosis of nerve cells by controlling the glutamate *N*‐methyl‐*D*‐aspartate (NMDA) pathway.	Muscaritoli 2021 Robberecht et al. 2020
Zinc	Depression, ADHD	It modulates cytokine activity and affects neurogenesis by influencing brain‐derived neurotrophic factor (BDNF) levels. It regulates the transport of dopamine. It is utilized as a cofactor by enzymes necessary for neurotransmitter, melatonin, and prostaglandin metabolism.	Grajek et al. 2022 Lai et al. 2012 Robberecht et al. 2020
Omega‐3	Depression	It influences cellular membrane function and intracellular signaling pathways, may improve white matter integrity, regulates HPA axis hyperactivity, can modulate brain‐derived neurotrophic factor, affects the production and activity of neurotransmitters, reduces oxidative stress, decreases neuroinflammation through its anti‐neurodegenerative activity, and has a positive effect on synapses.	Kong et al. 2025 Campisi et al. 2024 Serefko et al. 2024 Gui et al. 2024 Thakur et al. 2023 Reily et al. 2023
Probiotic	Depression, anxiety, ADHD	Reduction of the effects of inflammation and oxidative stress, sufficient production of amino acids and fatty acids utilized in the synthesis of neurochemicals necessary for the individual's positive emotional state (mood), production of certain bioactive molecules (peptides and hormones) that directly or indirectly regulate host behavior, and influence on the composition of the gut microbiota.	Gayathri and Rashmi 2017 Kalenik et al. 2021
Prebiotic	Depression, anxiety	It exhibits a neuromodulatory effect. It possesses a probable bifidogenic effect. It reduces inflammation.	Zhao et al. 2023 Radford‐Smith and Anthony 2023

## 4 Methods

### 4.1 Search Strategy and Narrative Synthesis Approach

To provide a comprehensive narrative synthesis of current evidence, a structured literature search was conducted across PubMed, Web of Science, and Scopus databases. The search encompassed English‐language original research without time limitations. The initial strategy utilized a broad set of terms to ensure maximal coverage of the intersection between ASD and nutrition. Primary search strings combined “autism,” “autistic,” “ASD,” or “autism spectrum disorder” with terms including “dietary,” “diet,” “nutrition,” “vitamin,” “mineral,” “probiotics,” and “omega‐3” (the conceptual framework is outlined in Table 2).

**TABLE 2:** Keywords and search parameters used for literature identification.

autism OR autistic OR ASD OR autism spectrum disorder	AND	dietary OR diet OR nutrition OR vitamin OR ‘vitamin D’ OR ‘Vitamin E’ OR ‘vitamin C’ OR ‘vitamin A’ OR ‘vitamin B12’ OR ‘vitamin B6’ OR ‘folic acid’ OR riboflavin OR thiamine OR niacin OR mineral OR iron OR zinc OR calcium OR magnesium OR potassium OR selenium OR iodine OR phosphorus OR copper OR probiotics OR prebiotics OR omega‐3 OR antioxidants OR fiber OR ‘coenzyme Q_10_’ OR supplement
Manual screening filters (targeted mental health and behavioral outcomes)		To ensure full replicability, the manual screening phase consistently targeted the following operational terms within the literature: mental health, behavioral problems, irritability, hyperactivity, stereotypic behavior, inattention, anxiety, depression, and aggression.

### 4.2 Selection Process and Manual Screening

Given that psychiatric and behavioral comorbidities in ASD are often inconsistently indexed in electronic databases, we employed a two‐stage screening process designed for high sensitivity. Following an initial broad electronic search, we performed a comprehensive manual screening of titles, abstracts, and methodology sections. Rather than relying solely on automated keyword filters, we manually verified whether each study utilized standardized psychometric instruments to quantify mental health symptoms. During this manual screening stage, a predefined set of target mental health and behavioral descriptors was consistently applied as operational filters to determine study inclusion (these specific manual screening terms are now explicitly detailed in Table 2 to ensure full methodological transparency and reproducibility. This “hand‐searching” approach ensured the inclusion of relevant studies where psychiatric symptoms were rigorously evaluated as secondary outcomes, even if not listed as primary keywords.

To ensure the credibility of the synthesis, a structured quality appraisal was performed for each included study. Methodological rigor—including considerations of blinding, sample size, and adherence monitoring—is explicitly summarized in the “methodological notes” column of the respective tables.

### 4.3 Eligibility Criteria and Conceptual Definitions

This synthesis included human studies evaluating the relationship between nutritional factors and mental health outcomes in individuals with ASD. For the purposes of this review, symptoms such as irritability, hyperactivity, stereotypic behavior, and inattention were categorized as “mental health‐related problems.” While these often overlap with the neurodevelopmental profile of ASD, we focused on them when they represented behavioral comorbidities that impair daily functioning, rather than core social‐communication deficits. This classification aligns with the subscales of standardized instruments used in the included studies (e.g., ABC, CBCL), which clinically distinguish these behaviors from primary diagnostic criteria.

### 4.4 Exclusion Criteria

Studies were excluded if they (1) involved animal models, review papers, or single case studies; (2) assessed the impact of nutrition exclusively on the core symptoms of autism (social interaction and communication); or (3) failed to employ validated assessment instruments (e.g., ABC, GARS, CBCL) to quantify mental health symptoms, thereby ensuring the methodological rigor of the synthesized evidence.

## 5 Results

A total of 21 studies were included in this review (Tables 3, 4, 5). Among these, one study included both children and adults, whereas the remaining 20 focused exclusively on children and/or adolescents. The included research explored various interventions, including omega‐3 (*n* = 5), vitamin D3 (*n* = 4), the combination of omega‐3 and vitamin D3 (*n* = 1), probiotic (*n* = 2), and prebiotic (*n* = 1) supplementation. Additionally, the review covered comprehensive nutrition and dietary interventions (*n* = 1), gluten‐free and casein‐free diets (GFCF) (*n* = 4), the low fermentable oligo‐, di‐, monosaccharides, and polyols (FODMAP) diet (*n* = 1), gluten‐ and dairy‐free diets (*n* = 1), and an assessment of manganese and zinc status (*n* = 1). To evaluate the mental health outcomes of individuals with autism, several standardized instruments were utilized across the studies, specifically the ABC, CBCL, CPRS‐R, ADHD‐IV, ADHD Rating Scale, ABC‐C, ABC‐2, and ABC‐J. The primary mental health parameters assessed were irritability, hyperactivity, stereotypic behavior, inattention, anxiety, ADHD, and depression.

**TABLE 3:** Summary of the studies showing the effects of diet interventions on mental health in individuals with autism spectrum disorder.

Author, year	Study design	Participants/duration	Nutritional intake/nutritional intervention	Mental health instruments	MH parameters	Results	Methodological notes
Whiteley et al. 2010	Randomized, controlled, single blind trial	Age = 4–12 years GFCF group = 26 Control group = 29 Duration = 12 months	Gluten‐free and casein‐free diet	ADHD‐IV	Inattention Hyperactivity	Significant improvement in inattention and hyperactivity scores at the 12‐month follow‐up.	–Lack of double‐blinding: High risk of observer bias and placebo effects due to parental awareness. –Adaptive design: Control group started the diet after 12 months, limiting long‐term comparative analysis. –Significant attrition: High dropout rate at 24 months, affecting the reliability of long‐term findings. –Confounding factors: Simultaneous use of multivitamin/calcium supplements. –Subjective outcomes: Core data relied on parent‐report scales (ADHD‐IV).
Johnson et al. 2011	Randomized controlled trial	Age = 3–5 years GFCF = 8 Healthy diet = 14 Duration = 3 months	Gluten‐free and casein‐free diet	CBCL	Anxious/depressed Inattention ADHD Anxiety	Significant reduction in ADHD subscale scores at 3‐month follow‐up; nonsignificant reductions observed in anxiety and anxious/depressed subscales.	–Small sample size (*n* = 22) with unequal group distribution. –Lack of double‐blinding: Single‐blind design prone to observer bias and placebo effect. –Short duration (3 months). –Adherence monitoring: Reliant on subjective 24‐h recalls and parental reports.
Pedersen et al. 2014	Randomized case‐control study	Age = 4–12 years Dietary intervention group = 38 Control group = 34 Duration = 12 months	Gluten‐free and casein‐free diet	ADHD‐IV	Inattention Hyperactivity	Most significant improvements in inattention and hyperactivity were observed in children aged 7–9 years.	–High attrition (37.5%). –Lack of blinding (risk of placebo effect). –Potential selection bias (*n* = 27 sub‐analysis). –Inconsistent baseline timing. –Male‐only sample.
Navarro et al. 2015	Randomized, double‐blind, placebo‐controlled	Age = 4–7 years GD (−) diet group = 6 GD (+) diet group = 6 Duration = 4 weeks	Gluten‐ and dairy‐free diet	ABC CBCL CPRS‐R	Hyperactivity Irritability Inattention	Hyperactivity: Significant reduction in the GFCF group versus an increase in the gluten/dairy‐containing group (ABC and CPRS‐R). Irritability: Nonsignificant decrease in the GFCF group compared to an increase in the gluten/dairy‐containing group. Inattention: Reductions observed in both groups based on CBCL and CPRS‐R scores.	–Small sample size (*n* = 12). –Attrition risk (incomplete follow‐up data). –Reliance on parental reporting (potential placebo effect). –Short intervention duration.
González‐Domenech et al. 2019	Cross‐over clinical pilot trial	Age = 3–18 years Group A = 12 Group B = 16 Duration = 6 months	Gluten‐free and casein‐free diet	ABC	Irritability Hyperactivity Stereotypic behavior	The GFCF diet demonstrated no significant impact on irritability or hyperactivity scores.	–Underpowered sample size (*n* = 28). –Lack of double‐blinding (potential placebo effect). –No washout period (potential carry‐over risk). –Wide age range (3–18 years). –Subjective outcomes based on parental reports (ABC). –Imprecise dietary adherence monitoring via urinary beta‐casomorphin.
Nogay et al. 2021	Randomized controlled pilot trial	Age = 6–17 years Low FODMAP diet group = 7 Control group (habitual diet) = 8 Duration = 2 weeks	Low FODMAP diet	ABC	Irritability Hyperactivity Stereotypic behavior	In the low FODMAP group, scores for irritability, stereotypic behavior, and hyperactivity showed nonsignificant reductions at follow‐up compared to baseline. Conversely, the control group demonstrated a significant increase in hyperactivity scores. While the low FODMAP cohort exhibited lower scores across all three domains compared to the control group at the 2‐week mark, these intergroup differences did not reach statistical significance.	–Small sample size (pilot study) and short duration (2 weeks). –Lack of blinding: Open‐label design prone to placebo effect and observer bias. –Subjective assessments: Results based entirely on parental reports and short food diaries. –Potential confounding: Inability to isolate the effects of reduced gluten intake from the low FODMAP diet.

Abbreviations: ABC, Aberrant Behavior Checklist; ADHD‐IV, attention‐deficit hyperactivity disorder—IV scale; CBCL, Child Behavior Checklist; CPRS‐R, Conners Parent Rating Scale‐Revised; GD (‐) diet, gluten‐dairy‐free diet; GD (+) diet, gluten‐dairy‐containing diet; MH, mental health.

**TABLE 4:** Summary of the studies showing the effects of vitamins and minerals on mental health in individuals with autism spectrum disorder.

Author, year	Study design	Participants, duration	Nutritional intake/ nutritional intervention	Mental health instruments	MH parameters	Results	Methodological notes
Saad et al. 2016	Case‐control, cross‐sectional study	Age = 3–9 years ASD = 122 TD = 100 Duration = 3 months	Vitamin D3 (300 IU/kg/day, not exceeding 5000 IU/day) was given to 106 children with ASD with low serum 25‐OH D levels (< 30 ng/mL).	ABC	Irritability Hyperactivity Stereotypic behavior	Vitamin D supplementation led to significant improvements in irritability (*p* = 0.01), hyperactivity (*p* = 0.021), and stereotypic behavior (*p* = 0.04) subscale scores among children with ASD.	–Open‐label design: High risk of observer bias due to lack of a placebo control group. –Non‐blinded assessment: Parents and clinicians are aware of treatment, potentially skewing subjective scores. –Short‐term follow‐up (3 months) without long‐term monitoring.
Kerley et al. 2017	Double‐blind, randomized, placebo‐controlled trial	Age = 3–11 years ASD = 42 VID = 22 PL = 20 Duration = 20 weeks	Vitamin D3 group received 2000 IU vitamin D3/day	ABC	Irritability Hyperactivity Stereotypic behavior	Vitamin D supplementation yielded no significant changes in irritability, hyperactivity, or stereotypic behavior subscale scores.	–Small sample size. –Included vitamin D‐sufficient children (60%, > 50 nmol/L), potentially diluting effects. –Higher mean age (7.9 years) may limit neurodevelopmental response. –Reliance on subjective parental reports for behavioral outcomes. –Did not account for VDR polymorphisms or binding proteins.
Mazahery et al. 2019	Randomized controlled trial	Age = 2.5–8 years VID = 30 OM = 28 VIDOM = 25 PL = 28 Duration = 12 months	VID = 2000 IU/day OM = 722 mg DHA/day VIDOM = 2000 IU + 722 mg DHA/day	ABC	Irritability Hyperactivity Stereotypic behavior	Irritability scores significantly decreased in the vitamin D, omega‐3, and combined (vitamin D + omega‐3) groups compared to placebo. Hyperactivity also showed a significant reduction in the vitamin D group (*p* = 0.047), whereas no significant changes were observed in stereotypic behaviors across any intervention group.	–Small sample size for a four‐arm study; potentially underpowered. –Attrition bias: Higher dropout rate among children with more severe symptoms. –Limited generalizability due to strict exclusion of early developmental delays. –Technical limitation: Vitamin D assay was not DEQAS certified. –Non‐fasted blood draws may affect biochemical precision.
Jayanath et al. 2021	Cross‐sectional study	Age = 3–18 years ASD = 103 Duration = 3 months	1200 IU vitamin D3/day (*n* = 20, children with ASD with vitamin D deficiency)	ABC‐2	Irritability Hyperactivity and noncompliance Stereotypic behavior	Vitamin D supplementation yielded nonsignificant reductions in irritability, hyperactivity, and stereotypic behavior subscale scores.	–Nonrandomized intervention: Treatment provided only to a deficient subgroup without a formal control group. –Small sample size for the follow‐up phase. –Risk of observer bias: Parents knew the treatment status while completing the ABC‐2. –Subjective assessment of sun exposure and physical activity (recall bias).
Hawari et al. 2020	Case‐control study	Age = 3–12 years ASD = 31 ADHD = 29 ASD‐C = 11 TD = 30	Manganese was measured in whole blood, and zinc in serum	ADHD rating scale	Inattention Hyperactivity	No associations were observed between metal levels and ASD‐C scores. The strongest correlation was found for manganese in the ADHD group. Furthermore, serum zinc levels remained within the normal clinical range across all groups.	–Small sample size (*n* = 101 total; only 11 comorbid cases). –Case‐control design: Shows correlation but not causality. –Limited generalizability: Single‐center study in Damascus. –Subjective assessments: Heavy reliance on parental reports for history and behavioral symptoms. –Environmental confounding: Potential sources of metal exposure not fully analyzed.
Javadfar et al. 2020	Randomized, double‐blind, placebo‐controlled, parallel‐group	Age = 3–13 years VID = 26 PL = 26 Duration = 15 weeks	VID = 300 IU/kg daily up to a maximum of 6000 IU/day	ABC‐C	Irritability Hyperactivity Stereotypic behavior	In the vitamin D group, irritability scores significantly decreased (*p* = 0.001), whereas reductions in hyperactivity and stereotypic behaviors were nonsignificant. By the 15th week, no statistically significant differences were observed between the vitamin D and placebo groups across all three behavioral domains.	–Small sample size (*n* = 52) and short intervention period (15 weeks). –Geographic/cultural specificity: Results may be influenced by local dietary habits and sun exposure levels. –Single‐center study; limited generalizability beyond the local population. –Subjective dietary assessment via 3‐day records (potential reporting bias).

Abbreviations: ABC‐2, Aberrant Behavior Checklist Second Edition; ABC‐C, Aberrant Behavior Checklist–Community; DHA, docosahexaenoic acid; OM, omega‐3; PL, placebo; VID, vitamin D; VIDOM, vitamin D + omega‐3.

**TABLE 5:** Summary of the studies showing the effects of other nutritional interventions on mental health in individuals with autism spectrum disorder.

Author, year	Study design	Participants/ duration	Nutritional intake/ nutritional intervention	Mental health instruments	MH parameters	Results	Methodological notes
Amminger et al. 2007	Randomized, double‐blind, placebo‐controlled pilot study	Age = 5–17 years Intervention group = 7 PL group = 6 Duration = 6 weeks	Intervention group = 120 mg EPA and 100 mg DHA plus 1 mg of vitamin E/day	ABC	Irritability Hyperactivity Stereotypic behavior	Nonsignificant reductions were observed in irritability, hyperactivity, and stereotypic behavior scores following the 6‐week intervention.	–Very small sample size (*n* = 22) and short study duration (6 weeks). –No biochemical monitoring of plasma or red blood cell lipid levels. –Selection bias: Participants were preselected for high irritability scores only. –Relying on clinician ratings (ABC) without incorporating objective physiological or parent‐at‐home observations.
Bent et al. 2011	Randomized controlled pilot trial	Age = 3–8 years Intervention group = 14 PL group = 13 Duration = 12 weeks	Intervention group = 350 mg of eicosapentanoic acid (EPA) and 230 mg of docosahexanoic acid (DHA)/twice a day	ABC	Irritability Hyperactivity Stereotypic behavior	The omega‐3 group showed greater improvements in hyperactivity and stereotypic behaviors than the placebo group, though these differences did not reach statistical significance.	–Small sample size (*n* = 27). –“Floor effect” due to mild baseline hyperactivity levels in participants. –Reliance on subjective parent‐completed scales (ABC). –Compliance was measured via subjective parent recall rather than objective counts. –Potential confounding from the use of safflower oil as a biologically active placebo.
Bent et al. 2014	Randomized, controlled, double‐blind trial	Age = 5–8 years Intervention group = 29 PL group = 28 Duration = 6 weeks	İntervention group = 350 mg of eicosapentanoic acid (EPA) and 230 mg of docosahexanoic acid (DHA)/twice a day	ABC	Irritability Hyperactivity Stereotypic behavior	While the omega‐3 group showed greater improvements in hyperactivity compared to placebo, this difference was nonsignificant; however, a statistically significant improvement was observed in stereotypic behaviors.	–Internet‐based trial: No direct in‐person clinical observation or diagnosis verification. –Entirely dependent on subjective parent and teacher reports (risk of observer bias). No biochemical monitoring (blood fatty acid levels) to verify adherence. –Small sample size (*n* = 57) resulting in low statistical power. –Technical coding error in the automated participant screening system. –Short study duration (6 weeks).
Ooi et al. 2015	Open‐label pilot follow‐up trial	Age = 7–18 years ASD = 41 Duration = 12 weeks	840 mg DHA, 192 mg EPA, 66 mg arachidonic acid (AA), 144 mg gamma linolenic acid, 60 mg vitamin E, and 3 mg thyme oil/twice a day	CBCL	Anxious/depressed Attention problems Rule‐breaking behaviors Aggressive behaviors	Combined omega‐3 and omega‐6 supplementation led to improvements across anxious/depressed, attention, and aggressive behavior domains, though statistical significance was only reached for attention problems.	–Open‐label pilot study: High risk of placebo effect and observer bias. –Entirely dependent on subjective parent‐reported behavioral scales (CBCL). –Small sample size with significant gender imbalance (88% male). –No control group to account for effects of standard routine care. –Lack of long‐term follow‐up beyond 12 weeks.
Adams et al. 2018	Randomized, controlled, single‐blind trial	Age = 3–58 years ASD = 67 Treatment group = 37 Nontreatment group = 30 TD = 50 Duration = 12 months	For treatment group, Day 0: Vitamin/mineral supplementation begins. Day 30: Essential fatty acid supplementation begins. Day 60: Epsom salt baths begin. Day 90: Carnitine supplementation begins. Day 180: Digestive enzyme supplementation begins. Day 210: Healthy, casein‐free, gluten‐free diet begins.	ABC	Irritability Hyperactivity Stereotypic behavior	The treatment group demonstrated significantly greater improvements in irritability, hyperactivity, and stereotypic behavior scores compared to the nontreatment group.	–Single‐blind design: Evaluators were blinded, but participants/parents were not (high risk of placebo effect). –Confounding interventions: Impossible to isolate the effects of individual treatments (diet vs. multiple supplements). –Subjective assessment bias: Heavy reliance on parent‐reported scales susceptible to expectation bias. –Self‐evaluated dietary compliance; lack of strict objective monitoring for HGCSF diet.
Arnold et al. 2019	Randomized, placebo‐controlled, crossover pilot trial	Age = 3–12 years Probiotic‐placebo = 6 Placebo‐probiotic = 4 Duration = 19 weeks	The probiotic mix *L. casei*, *Lactobacillus plantarum*, *Lactobacillus acidophilus*, and *B. longum*, *Bifidobacterium infantis*, and *Bifidobacterium breve*, one strain of *S. thermophiles*	ABC	Irritability Hyperactivity Stereotypic behavior	Probiotic supplementation resulted in nonsignificant improvements across irritability, hyperactivity, and stereotypic behavior scores.	–Small sample size; no sex or GI‐type specific analysis. –Risk of carry‐over effects due to a short (3‐week) washout period. –Lack of metagenomic and metabolomic profiling for gut microbiota. –Entirely dependent on subjective parent‐reported behavioral and GI scales.
Inoue et al. 2019	Prospective, open‐label pilot trial	Age = 4–9 years ASD = 13 Duration = 2–15 months	PHGG (6 g/day)	ABC‐J	Irritability	PHGG supplementation led to a significant reduction in ABC‐J irritability subscale scores (*p* < 0.01). Furthermore, significant positive correlations (*p* < 0.05) were identified between irritability scores and the presence of *Streptococcus salivarius*, *Alistipes putredinis* (strain Marseille), *Bacteroides ovatus*, and *Clostridium indolis*.	–Small sample size (*n* = 13 for stool, *n* = 9 for blood). –Open‐label design without a placebo control group. –Potential for parental reporting bias in defecation frequency records. –Lack of strict dietary monitoring (e.g., total fiber and fluid intake) during the trial.
Sherman et al. 2022	Randomized, double‐blinded, placebo‐controlled trial	Age = 6–15 years ASD = 35 Probiotic = 18 Placebo = 17 Duration = 16 weeks	*Lactobacillus plantarum* PS128, 6 × 10^10^ CFUs per day	ABC‐2	Irritability Hyperactivity Stereotypic behavior	Baseline anti‐lysoganglioside GM1 levels positively correlated with ABC‐2 stereotypic behavior scores in the probiotic group, whereas no such association was observed in the placebo group.	–Small sample size (*n* = 35) with high dropout rates for biological samples. –High heterogeneity due to a wide age range (3–25 years). –Potential bias in parent ratings due to cultural and language barriers in minority groups. –Risk of Type I error (false positives) due to lack of adjustments for multiple comparisons. –Absence of a neurotypical control group for baseline biomarker comparison.
Boone et al. 2022	Randomized, double‐blinded, placebo‐controlled trial	Age = 18–38 months ASD = 31 Intervention group = 15 Placebo group = 16 Duration = 90 days	338 mg EPA, 225 mg DHA, 83 mg GLA, and 306 mg oleic acid/day	CBCL	Anxious/depressed Attention problems Rule‐breaking behaviors Aggressive behaviors Anxiety problems Attention deficit/ hyperactivity problems Oppositional defiant problems	The intervention group demonstrated a significant reduction in anxious/depressed behaviors with a moderate effect size compared to placebo. While improvements were also observed in attention and aggressive behaviors, CBCL scale assessments revealed no statistically significant intergroup differences regarding oppositional defiant problems.	–Small pilot study (*n* = 31) focused strictly on preterm infants with ASD symptoms. –Entirely reliant on subjective caregiver reports for behavior and sleep data. –Risk of Type I error due to multiple statistical comparisons. –Inconsistent reporting timeframes across different assessment tools.

In a 12‐month, randomized, controlled, single‐blinded study involving individuals with ASD aged 4–12 years, Whiteley et al. (2010) demonstrated that the GFCF dietary intervention significantly improved inattention and hyperactivity outcomes. Similarly, Pedersen et al. (2014) investigated the same diet within the same age group and duration, reporting a positive effect on inattention and hyperactivity domains; notably, this effect was most pronounced in children aged 7–9 years. Both studies utilized the ADHD‐IV as the primary mental health instrument. In a shorter, 3‐month intervention conducted by Johnson et al. (2011) among children with ASD aged 3–5 years, assessment via the CBCL revealed a statistically significant reduction in the ADHD subscale, while reductions in the anxious/depressed and anxiety subscales did not reach statistical significance. Conversely, a crossover clinical study by González‐Domenech et al. (2019) that employed the ABC instrument reported no significant effect of the GFCF intervention. Furthermore, Navarro et al. (2015) conducted a 4‐week, randomized, double‐blind, placebo‐controlled pilot study in which six children with ASD (aged 4–7 years) received a gluten‐ and dairy‐free diet, while a control group of six children consumed a gluten‐dairy–containing diet. The results indicated that dietary restriction reduced hyperactivity and irritability. However, inattentive behaviors decreased in both the intervention and control groups. This study utilized the CBCL, ABC, and CPRS‐R as mental health instruments (Navarro et al. 2015).

In a study involving children with ASD aged 6–17 years who met the Rome IV criteria for constipation and/or abdominal pain, participants were divided into two groups: one following a low FODMAP diet for 2 weeks and a control group maintaining their usual dietary habits. Although the findings did not reach statistical significance, the group following the low FODMAP diet exhibited lower scores for irritability, stereotypic behavior, and hyperactivity/noncompliance at the follow‐up assessment compared to baseline (Nogay et al. 2021).

In a study involving children with ASD who had low baseline serum 25‐OH‐D levels, weight‐based administration of vitamin D3 (300 IU/kg/day) for 3 months resulted in significant improvements in irritability, hyperactivity, and stereotypic behavior subscales (Saad et al. 2016). Another 3‐month trial investigated a fixed dose of 1200 IU/day; in the vitamin D‐deficient group, reductions in these subscales did not reach statistical significance. However, within the non‐deficient group, significant improvements were observed in hyperactivity and stereotypic behavior, while changes in irritability remained nonsignificant (Jayanath et al. 2021). Furthermore, two double‐blind, randomized, placebo‐controlled trials examined vitamin D supplementation with varying outcomes. Daily administration of 2000 IU of vitamin D3 for 20 weeks did not yield significant changes in behavioral subscales compared to baseline (Kerley et al. 2017). In the second trial, which utilized a weight‐based dose of 300 IU/kg/day for 15 weeks, a significant intragroup reduction in irritability was reported. However, by the conclusion of the 15th week, no statistically significant differences were observed between the vitamin D and placebo groups regarding irritability, hyperactivity, or stereotypic behavior scores (Javadfar et al. 2020).

In a 6‐week trial using the ABC as the primary mental health instrument, children with ASD (aged 5–17 years) were administered a supplement of 120 mg EPA and 100 mg DHA. The results showed nonsignificant reductions in irritability, hyperactivity, and stereotypic behavior subscales compared to the placebo group (Amminger et al. 2007). In a subsequent randomized controlled study, Bent et al. (2011) investigated a higher dosage (350 mg EPA and 230 mg DHA, twice daily) over 12 weeks. While the omega‐3 group showed greater improvement in hyperactivity and stereotypic behaviors than placebo, these differences did not reach statistical significance. However, in a similar study with a larger sample size conducted by the same authors, significant improvements in stereotypic behaviors were observed after 6 weeks of omega‐3 supplementation (Bent et al. 2014). The effects of combined fatty acid supplementation have also been explored with varying results. Ooi et al. (2015) found that 12 weeks of omega‐3 and omega‐6 supplementation in children aged 7–18 years yielded improvements in anxious/depressed symptoms, attention problems, and aggressive behaviors; notably, only the change in attention problems was statistically significant. In another trial among younger children (aged 18–38 months), a 90‐day intervention combining omega‐3, omega‐6, and omega‐9 resulted in a significant decrease in anxious/depressed behaviors, with a moderate effect size. While reductions in attention and aggressive behaviors were also observed, they remained statistically nonsignificant (Boone et al. 2022). Finally, a 12‐month study evaluating the synergy between omega‐3 and vitamin D reported that irritability significantly decreased in the vitamin D, omega‐3, and combined supplementation groups compared to the placebo. In contrast, no significant changes were detected in stereotypic behaviors across these groups (Mazahery et al. 2019).

In a randomized, double‐blind, placebo‐controlled trial conducted by Sherman et al. (2022), the administration of *Lactobacillus plantarum* PS128 (6 × 10^10^ CFUs) for 16 weeks resulted in a significant reduction in stereotypic behaviors. In contrast, a randomized, placebo‐controlled crossover pilot study utilizing different probiotic species reported only statistically nonsignificant improvements in irritability, hyperactivity, and stereotypic behaviors (Arnold et al. 2019). Regarding prebiotic interventions, daily supplementation with 6 g of partially hydrolyzed guar gum (PHGG) in 13 children with ASD (aged 4–9 years) led to a significant reduction in the ABC‐J irritability subscale (Inoue et al. 2019). Furthermore, Adams et al. (2018) investigated the effects of a comprehensive nutritional and dietary intervention in a randomized, controlled, single‐blind, 12‐month study involving 67 individuals with ASD compared to 50 neurotypically developing controls. The intervention protocol began with specialized vitamin/mineral supplementation and was gradually expanded to include essential fatty acids, carnitine, digestive enzymes, and a healthy, gluten‐free, casein‐free, and soy‐free diet (HGCSF diet). The results demonstrated significantly greater improvements in irritability, hyperactivity, and stereotypic behavior scores in the intervention group compared to the nontreatment group (Adams et al. 2018).

In a case‐control study investigating levels of manganese and zinc among children diagnosed with ASD, ADHD, and ASD co‐occurring with ADHD (ASD‐C), researchers measured whole‐blood manganese levels, along with serum zinc concentrations. The trial, which utilized the ADHD Rating Scale as the primary mental health instrument, reported that manganese levels were significantly lower in both the ASD and the hyperactive‐predominant ADHD (ADHD‐H) groups compared to the control group. Although the difference was not statistically significant, manganese levels in the ASD‐C group were also lower than those in the control group. Furthermore, no significant differences in zinc levels were detected across the clinical groups compared with the control group (Hawari et al. 2020).

## 6 Discussion

In this review, 21 studies were analyzed to evaluate the impact of nutrition on the mental health challenges observed in individuals with ASD. It has recently been recognized that the mental health problems associated with ASD are distinct comorbidities rather than merely symptoms of the core disorder (Matson and Goldin 2013). The co‐occurrence of ADHD with autism is associated with greater impairments in adaptive functioning, health‐related quality of life, and executive function compared to autism alone (Lai et al. 2019; Rao and Landa 2014). Similarly, the presence of anxiety in autistic individuals exacerbates core symptoms—including social impairments, sensory features, and repetitive behaviors—and can accelerate the development of depression. Furthermore, behaviors such as aggression, self‐injurious behavior, and oppositional tendencies can emerge or increase with the onset of depression. The mental health problems observed in autism often persist from childhood into adolescence, potentially leading to long‐term adverse effects on health and quality of life (Lai et al. 2019). In this context, behavioral markers such as irritability, hyperactivity, and stereotypic behaviors are increasingly recognized as clinical proxies for underlying emotional dysregulation and anxiety disorders in the ASD population (Mazefsky et al. 2013). Consequently, the use of standardized instruments, such as the ABC or CBCL, provides a robust framework to quantify these internalizing and externalizing mental health challenges.

Studies evaluating the nutritional status of individuals with ASD have demonstrated that this population often exhibits an inadequate intake of essential nutrients—including vitamin A, folate, vitamin C, vitamin B12, and iron—compared to neurotypical peers (Marí‐Bauset et al. 2017; Barnhill et al. 2018; Al‐Farsi et al. 2013). Furthermore, emerging research indicates that vitamin D, vitamin B12, vitamin C, folic acid, iron, magnesium, omega‐3 fatty acids, and probiotics may exert positive effects on mental health outcomes (Dionisie et al. 2025; Ding and Zhang 2022; Muscaritoli 2021; Mikkelsen et al. 2016; Kong et al. 2025). Despite these potential benefits, the majority of nutrition‐related ASD research has historically focused on core diagnostic symptoms rather than broader psychiatric comorbidities.

A substantial body of literature examines the effects of GFCF dietary interventions on gastrointestinal (GI) and core symptoms in individuals with ASD (Ghalichi et al. 2016; Hyman et al. 2016). The theoretical framework supporting this diet suggests that increased intestinal permeability allows gluten and casein peptides to enter the bloodstream, subsequently triggering opioid activity that may exacerbate behavioral challenges (Quan et al. 2022). In three of the studies reviewed, the GFCF intervention led to significant reductions in inattention, hyperactivity, and ADHD subscales; however, reductions in anxiety and depressive symptoms did not reach statistical significance (Whiteley et al. 2010; Pedersen et al. 2014; Johnson et al. 2011). The inconsistency in these results may be attributed to the use of different assessment scales (e.g., ADHD‐IV and CBCL) and the lack of screening for celiac disease or gluten/casein sensitivities among participants. Conversely, González‐Domenech et al. (2019) reported no significant effect of the GFCF diet on irritability and hyperactivity. This lack of findings may be explained by the exclusion of individuals with confirmed gluten or casein allergies, as well as the wide age range (3–18 years) of the cohort, which increases interindividual variability. In contrast, Navarro et al. (2015) observed reductions in hyperactivity and irritability in a small group of children following a gluten‐ and dairy‐free diet. Despite the limited sample size (*n* = 12), the study's power was enhanced by the rigorous exclusion of participants with preexisting food allergies, celiac disease, or inflammatory bowel diseases (IBD). Synthesizing these data, the discrepancy between the findings of González‐Domenech et al. (2019) and Navarro et al. (2015) suggests that GFCF efficacy may be contingent upon underlying GI sensitivities or opioid–peptide activity. Critically, the exclusion of individuals with allergies by González‐Domenech et al. (2019) contrasts with the positive outcomes reported in Navarro et al.’s (2015) trial, which likely captured a subset of children with genuine dietary intolerances. However, the interpretation of these results remains cautious due to small sample sizes and nonstandardized dietary protocols. These findings highlight the urgent need for precision nutrition approaches—identifying specific biomarkers and sensitivities before implementing restrictive diets—to achieve measurable improvements in mental health. Ultimately, GFCF interventions should not be viewed as a universal treatment but as a targeted strategy for specific subsets of the ASD population exhibiting clinical dietary intolerances.

FODMAPs exert physiological effects by increasing osmotic water retention in the small intestine, elevating gas production (specifically hydrogen and methane), and promoting excessive short‐chain fatty acid (SCFA) production. In individuals with visceral hypersensitivity, these processes can lead to abdominal pain, bloating, flatulence, and altered bowel habits (Ribichini et al. 2024; Bertin et al. 2024). The GI symptoms triggered by FODMAPs mirror those frequently observed in both IBD and ASD. While the low FODMAP diet is an established intervention in IBD management, its efficacy in the ASD population remains unproven (Shindler et al. 2020). In a study investigating a 2‐week low‐FODMAP intervention on GI and behavioral symptoms in children with ASD, assessment using the ABC instrument showed lower scores for irritability, stereotypic behavior, and hyperactivity than in the control group; however, these findings did not reach statistical significance (Nogay et al. 2021). The authors attributed the lack of significance to the limited sample size and the brief duration of the intervention. Furthermore, it remains unclear whether the observed behavioral trends were influenced by the total FODMAP reduction, a specific FODMAP subtype, or the concurrent decrease in gluten intake inherent to the diet (Nogay et al. 2021). While research has explored the impact of FODMAPs on anxiety and depression in individuals with irritable bowel syndrome (IBS), their specific effects on these psychiatric comorbidities in ASD have yet to be investigated (Eswaran et al. 2017).

Vitamin D deficiency is recognized as a potential environmental risk factor for ASD (Kerley et al. 2017; Holick 2007). Current hypotheses suggest two primary mechanisms by which vitamin D influences ASD development: its role in immune regulation and neurodevelopment, and its involvement in gene regulation (Jayanath et al. 2021; Kočovská et al. 2012). Additionally, vitamin D is thought to mitigate autism severity by raising the seizure threshold, increasing T‐regulatory cells, protecting mitochondrial function, and upregulating glutathione, which facilitates the clearance of oxidative byproducts and the chelation of heavy metals (Cannell and Grant 2013). Despite the high prevalence of inadequate vitamin D intake and low serum levels frequently reported in children with ASD, serum 25(OH)D deficiency persists due to limited sunlight exposure, dietary insufficiencies, or impaired metabolic conversion (Hyman et al. 2012; Bala et al. 2016; Tostes et al. 2012; Bener et al. 2014; Patrick and Ames 2014).

The efficacy of vitamin D interventions must be evaluated in the context of baseline 25(OH)D status and achieved serum concentrations. Saad et al. (2016) administered a weight‐based dose (300 IU/kg/day) for 3 months to children with ASD who had low baseline levels (< 30 ng/mL), resulting in significant improvements in irritability, hyperactivity, and stereotypic behaviors. In this trial, 61% of participants achieved serum levels above 30 ng/mL, suggesting that clinical efficacy may be contingent upon reaching a therapeutic threshold in individuals with clear baseline deficiencies. In contrast, Jayanath et al. (2021) investigated a fixed dose of 1200 IU/day; interestingly, while the deficient group showed nonsignificant changes, the non‐deficient group exhibited significant improvements in hyperactivity and stereotypic behaviors. The biological basis for these improvements in non‐deficient individuals remains largely unexplained (Jayanath et al. 2021).

Despite these promising signals, the broader evidence for vitamin D remains fragmented. In a placebo‐controlled trial, Kerley et al. (2017) administered 2000 IU of vitamin D3 daily for 20 weeks but observed no significant behavioral changes compared to the placebo group. Unlike the study by Saad et al. (2016), participants in Kerley's trial had higher mean baseline 25(OH)D levels (approx. 51–58 nmol/L) and received a fixed rather than weight‐based dose, which may explain the discrepancy. Similarly, Javadfar et al. (2020) reported that, despite significant increases in serum 25(OH)D levels after 15 weeks, supplementation did not yield statistically significant improvements in mental health compared with placebo. These conflicting findings underscore that vitamin D supplementation is not a universal solution; its efficacy appears highly dependent on addressing biochemical deficiencies, employing precise dosage protocols (weight‐based vs. fixed), and accounting for individual metabolic variability.

Omega‐3 fatty acids, specifically eicosapentaenoic acid (EPA) and docosahexaenoic acid (DHA), play a critical role in the structural and functional development of the brain. Both EPA and DHA, alongside the omega‐6 fatty acid gamma‐linolenic acid (GLA), possess potent anti‐inflammatory properties. DHA, in particular, is essential for neurotransmitter function, synaptogenesis, gene expression, membrane fluidity, neurogenesis, neuroplasticity, and the mitigation of neuroinflammation (Boone et al. 2022; Simopoulos 2002; Janssen and Kiliaan 2014). The therapeutic impact of omega‐3 appears to be strongly influenced by the specific EPA/DHA dosage and the duration of the intervention. Amminger et al. (2007) demonstrated that administering 120 mg of EPA and 100 mg of DHA for 6 weeks to children with ASD (aged 5–17 years) led to nonsignificant reductions in irritability, hyperactivity, and stereotypic behaviors. Bent et al. (2011) conducted a similar trial utilizing a higher dosage (350 mg EPA and 230 mg DHA, twice daily) over a longer duration (12 weeks), finding that omega‐3 supplementation led to greater—though still statistically nonsignificant—improvements in hyperactivity and stereotypic behaviors compared to placebo. Conversely, in a subsequent study with a larger sample size, Bent et al. (2014) reported significant improvements in stereotypic behaviors using the same dosage protocol. This suggests that clinical signals in omega‐3 trials may be dose‐dependent and may only emerge with sufficient statistical power and the extended treatment windows necessary for neuronal membrane incorporation.

Two studies on this topic, which differed in duration, participant age, and sample size, both revealed reductions in anxious/depressed symptoms, attention problems, and aggressive behaviors (Ooi et al. 2015; Boone et al. 2022). While these findings highlight a consistent trend toward improvement, many results failed to reach statistical significance, particularly in smaller pilot trials. The heterogeneity in outcomes across these studies can be analyzed through the lens of varying EPA/DHA ratios and intervention durations, ranging from 6 to 12 weeks, further complicating the interpretation of clinical efficacy. Although the underlying mechanisms are not fully elucidated, the effects of omega‐3 are thought to involve the modulation of serotonergic and dopaminergic neurotransmission (Amminger et al. 2007; Hibbeln et al. 1998). Additionally, it has been proposed that the DHA or EPA–ARA ratio may regulate aggression by suppressing the noradrenergic system (Amminger et al. 2007; Hamazaki et al. 2005).

In a trial where omega‐3 and vitamin D were coadministered, the combined supplementation resulted in nonsignificant reductions in irritability and hyperactivity compared to placebo (Mazahery et al. 2019). This lack of statistical significance, likely due to small sample sizes, underscores that while these nutrients possess anti‐inflammatory and immunomodulatory properties, their impact on mental health comorbidities in ASD remains preliminary (Mazahery et al. 2017; Mazahery et al. 2016). Therefore, future studies with larger cohorts and standardized dosage protocols are essential. Synthesizing the results from vitamin D and omega‐3 interventions, it appears that these nutrients may act synergistically on the neuroendocrine system. While vitamin D modulates serotonin synthesis via the tryptophan hydroxylase 2 (TPH2) gene, omega‐3 fatty acids enhance membrane fluidity and dopaminergic signaling. Together, these mechanisms might provide a theoretical basis for the emotional stability observed in some trials; however, the clinical evidence remains inconsistent due to methodological variations in sample characteristics and dose–response relationships (Patrick and Ames 2014).

GI problems—such as constipation, abdominal pain, and diarrhea—are frequently observed in children with ASD and are thought to contribute significantly to problematic behaviors (Tan et al. 2021; Ferguson et al. 2019). To alleviate these behavioral challenges, research has increasingly focused on potential treatments that modulate the microbiota–gut–brain axis, with probiotics and prebiotics among the primary interventions (Tan et al. 2021). The efficacy of such interventions, however, must be evaluated in light of strain specificity and participants' baseline GI phenotypes.

In one of the reviewed probiotic studies, administration of *Lactobacillus plantarum* PS128 (6 × 10^10^ CFUs) for 16 weeks resulted in a reduction in stereotypic behaviors (Sherman et al. 2022). This finding suggests that high‐potency, strain‐specific interventions may be necessary to elicit neurobehavioral changes. In contrast, the study by Arnold et al. (2019), which utilized different probiotic species and varying dosage protocols, reported statistically nonsignificant improvements in irritability and hyperactivity. While these initial results are encouraging, the marked differences in probiotic strains, CFU counts, and trial durations make it difficult to generalize findings. Furthermore, the limited sample sizes across these trials increase the risk of individual variability overshadowing the true treatment effects.

Prebiotics are defined as substrates that are selectively utilized by host microorganisms to confer a health benefit (Azari et al. 2024). PHGG is a water‐soluble dietary fiber with prebiotic properties that is hypothesized to positively modulate the gut microbiota (Ohashi et al. 2015). A study on this topic demonstrated that daily supplementation with 6 g of PHGG led to a significant reduction in the ABC‐J irritability subscale, alongside an increase in defecation frequency (Inoue et al. 2019). Although the sample size was small, the prebiotic intervention reduced irritability, specifically by alleviating symptoms in children with a constipation‐predominant GI phenotype. The observed improvements in irritability following prebiotic and probiotic interventions reinforce the gut–brain axis hypothesis (Inoue et al. 2019; Sherman et al. 2022). Alleviating physical discomfort—such as constipation via PHGG—appears to have a direct downstream effect on mental health behaviors, suggesting that GI health is an inseparable component of psychiatric stability in ASD. Consequently, future trials should stratify participants based on their GI symptomatology to better identify which subsets of the ASD population respond most effectively to specific microbiota‐modulating agents. However, more rigorously controlled, large‐scale studies remain necessary to validate this relationship.

Zinc is an essential metal that plays a critical role in the structural and functional integrity of the brain (Black 1998). Deficiencies in cerebral zinc levels have been linked to pathological conditions, including ADHD. Given that dopamine and melatonin are central to the pathophysiology of ADHD, and considering that melatonin synthesis is zinc‐regulated, zinc deficiency is regarded as a significant factor in ADHD etiology (Talebi et al. 2022; Dodig‐Curkovic et al. 2009). Manganese is equally vital for neurological health, providing neuroprotection through its antioxidant properties (DRI 2005). While manganese is abundant in various foods—making deficiency rare—current evidence suggests that excessive exposure may impair neurodevelopmental mechanisms, including oxidative stress pathways, neuroinflammation, the gut–brain axis, and mitochondrial function (Aschner et al. 2024; Martins et al. 2020). Hawari et al. (2020) investigated the levels of zinc and manganese in children with ASD, ADHD, and co‐occurring ASD‐ADHD (ASD‐C) in Syria. The study revealed that manganese levels in both the ASD and ADHD groups were significantly lower than those in the control group. While manganese levels in the ASD‐C group were also reduced, this finding did not reach statistical significance. In contrast, serum zinc levels remained within normal ranges across all three diagnostic groups (Hawari et al. 2020). The authors noted that the observed low manganese levels—which contradict some previous literature—may be attributed to regional variations in the manganese content of drinking water, genetic polymorphisms in the manganese transporters *SLC30A10* and *SLC39A8*, and the potential impact of local socioeconomic conditions on child nutrition (Yousef et al. 2011; Sanders et al. 2015).

Adams et al. (2018) investigated a multifaceted nutritional and dietary protocol in both children and adults with ASD. Building on previous short‐term research indicating specific clinical benefits, the intervention synergistically combined various vitamins and minerals, omega‐3, ‐6, and ‐9 fatty acids, L‐carnitine, and a HGCSF diet. The trial demonstrated significantly greater improvements in irritability, hyperactivity, and stereotypic behavior scores within the intervention group compared to the nontreatment cohort. However, implementing such a combined therapeutic approach introduces a major methodological limitation: It remains impossible to isolate the individual efficacy or the specific contribution of any single component. As emphasized by Adams et al. (2018), while these integrated strategies are promising, further comprehensive research is essential to establish standardized dosages and rigorously evaluate potential side effects before such broad‐spectrum interventions can be formally recommended in clinical practice.

## 7 Limitations

Several limitations of this review warrant consideration. First, the absence of a distinct clinical boundary between core ASD behavioral symptoms and co‐occurring mental health challenges often results in the use of divergent assessment scales across the literature. This psychometric heterogeneity, frequently compounded by small and underpowered sample sizes, complicates the isolation of specific nutritional effects on distinct mental health outcomes. Second, while the literature search was robust—encompassing PubMed, Web of Science, and Scopus—the exclusion of specialized databases such as PsycINFO may have precluded the identification of additional psychologically focused trials. Furthermore, because this study was designed as a narrative synthesis rather than a systematic review, the literature search relied partially on manual screening filters based on predefined operational descriptors, which may inherently limit the complete reproducibility of the search pipeline. Third, the brief duration of numerous included studies (some ranging from 2 to 6 weeks) is likely insufficient to adequately evaluate the long‐term clinical efficacy or safety profiles of the investigated supplements. Fourth, although the methodological quality, sample sizes, and procedural constraints of the included primary studies were qualitatively appraised and detailed within the text and tables (specifically under the “methodological notes” columns), a formal, standardized risk‐of‐bias assessment tool was not utilized. The lack of such a structured scoring matrix represents a limitation that should be considered when interpreting the generalizability of the synthesized evidence. Finally, the significant variability in dosage protocols and the prevalence of statistically nonsignificant findings across multiple trials underscore that the current evidence remains inconsistent. Consequently, these results should be interpreted as preliminary and addressed with caution in clinical contexts.

## 8 Conclusion

In this narrative synthesis, 21 studies were evaluated to elucidate the relationship between nutritional interventions and mental health problems in individuals with ASD. Overall, the findings across the reviewed literature remain inconsistent and provide only preliminary signals of therapeutic benefit. While several trials suggest that vitamin D3, omega‐3 fatty acids, and specific probiotic strains may alleviate symptoms such as irritability and anxiety, significant methodological gaps—including small sample sizes, brief intervention durations, and the use of diverse psychometric instruments—preclude a definitive consensus. In light of the current evidence, these nutritional interventions cannot be classified as established treatments, and the available data are insufficient to support the formulation of universal clinical guidelines. The clinical priority for this population should remain the prevention of nutrient deficiencies through individualized and balanced nutrition. Formal recommendations for routine supplementation must await the results of future large‐scale, standardized, and rigorously controlled trials capable of confirming clinical efficacy and establishing long‐term safety profiles.

## Author Contributions

**Nalan Hakime Nogay**: writing – review and editing. **Gulin Ozturk Ozkan**: writing – review and editing.

## Funding

The authors have nothing to report.

## Conflicts of Interest

The authors declare no conflicts of interest.

## Data Availability

Data sharing is not applicable to this article as no datasets were generated or analyzed during the current study.
